# A One-Dimensional Effective Model for Nanotransistors in Landauer–Büttiker Formalism

**DOI:** 10.3390/mi11040359

**Published:** 2020-03-30

**Authors:** Ulrich Wulf

**Affiliations:** Department of Computational Physics, Brandenburg University of Technology Cottbus-Senftenberg, PO box 101344, 03013 Cottbus, Germany; ulrich.wulf@b-tu.de

**Keywords:** nanotransistor, quantum transport, Landauer–Büttiker formalism, R-matrix method

## Abstract

In a series of publications, we developed a compact model for nanotransistors in which quantum transport in a variety of industrial nano-FETs was described quantitatively. The compact nanotransistor model allows for the extraction of important device parameters as the effective height of the source-drain barrier, device heating, and the quality of the coupling between conduction channel and the contacts. Starting from a basic description of quantum transport in a multi-terminal device in Landauer–Büttiker formalism, we give a detailed derivation of all relevant formulas necessary to construct our compact nanotransistor model. Here we make extensive use of the the R-matrix method.

## 1. Introduction

Around 2005–2010, the transistors obeying Moore’s law where strained high-k metal gate MOSFETs with channel lengths between 20–40 nm. At this point a further reduction of the transistor size in a conventional MOSFET becomes difficult because of short channel effects that reduce the gate voltage control over the conduction channel. To counteract this loss of control new transistor architectures were developed. In industrial applications the FinFET and the SOI transistor architecture hwere applied to continue Moore’s law to presently below 10nm gate length. It is now generally accepted that in this length-regime quantum transport becomes dominant and Moore’s law thus enters the domain of quantum electronics.

In a series of papers [1,2,3,4,5,6,7,8], we developed a compact transistor model in which quantum transport in a variety of industrial nano-FETs could be described quantitatively [6,7,8]. Our compact transistor model allows for the extraction of important device parameters as the effective height of the source-drain barrier of the transistor, device heating, and the overlap between the wave functions in the contacts and in the electron channel thus describing the quality of the coupling between conduction channel and contacts. Our starting point is a general description of quantum transport in a multi-terminal device in Landauer–Büttiker formalism which we formulate in the R-matrix formalism [1,2]. Using the R-matrix formalism as the essential tool, we give in this paper a systematic and comprehensive derivation of all relevant formulas necessary to construct our compact transistor model.

The concept of Landauer–Büttiker formalism was pioneered by Frenkel [9], Ehrenberg and Hönl [10], Landauer [11,12], Tsu and Esaki [13], Fisher and Lee [14], and Büttiker [15,16,17]. The central quantities of Landauer–Büttiker formalism are the transmission coefficients of the scattering solutions of the Schrödinger equation. In recent decades, Landauer–Büttiker formalism has been applied in fundamental research to numerous mesoscopic systems. Well-known examples include interferometric measurements in an Aharonov-Bohm ring [15,18], the quenching of the quantum Hall effect in small junctions [19,20], the quantized conductance in ballistic point contacts [21,22], resonant transport through double barrier systems [23], Coulomb blockade oscillations [24,25], spintronic effects [26,27,28], and Hanbury Brown and Twiss experiments on current fluctuations [29,30,31,32].

For formal developments as well as for numerical- and analytical evaluations of the mentioned transmission coefficients of the scattering functions we employ the R-matrix method. This method was introduced by Wigner and Eisenbud and has been widely used in atomic and nuclear physics (for reviews see Refs. [33,34]). A similar method was developed by Kapur and Peierls [35]. The application of the R-matrix technique to mesoscopic semiconductor systems was demonstrated by Smrčka [36] for one-dimensional structures. Since then it has been applied to a variety of other semiconductor nano-structures as point contacts [37], quantum dots [38,39], resonant tunneling in double barrier systems [40], four-terminal cross-junctions [41], gate all around and double gate MOSFETs [42,43], nanowire transistors [44], spin FETs [45], magneto-transport in nanowires [46], ballistic transport in wrinkled superlattices [47], and spin controlled logic gates [48]. A conceptual advantage of the R-matrix method is that for the construction of the transmission coefficients only properties of general wave function solutions of the time-independent Schrödinger equation are necessary (see Equation (21)). This is in contrast to the often used non-equillibrium Green’s function approach [49] which relies on the calculation of Green’s functions from which the transmission coefficients have to be calculated via the Fisher-Lee relation [14]. Moreover, the existence of the discrete representation of the R-matrix in the eigenbasis of the Wigner–Eisenbud functions (see Equation (22)) allows for the systematic construction of the one-dimensional effective transistor model used in Refs. [6,7,8] as will be described in Section 5, Section 6, Section 7 and Section 8.

## 2. Landauer–Büttiker Formula for Multi-Terminal Devices

**Figure 1:**
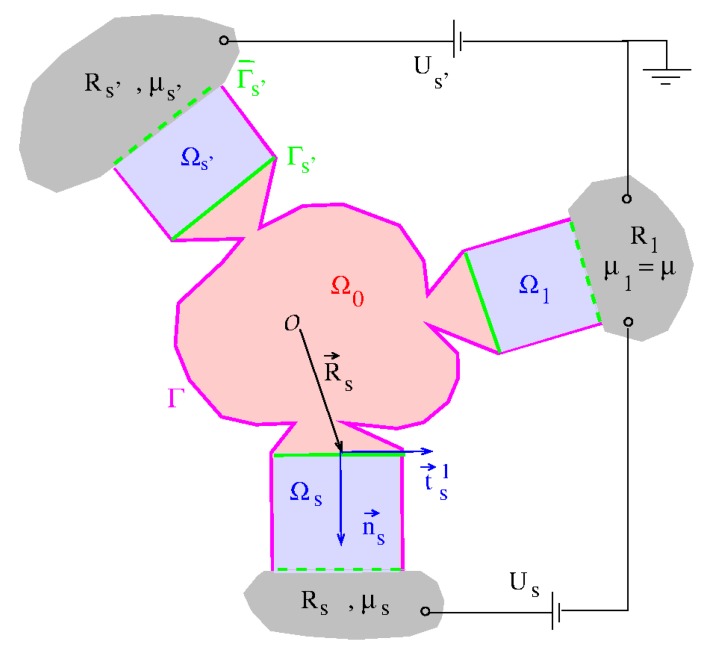
Idealized multi-terminal system: N=3 terminals denoted with the index *s* are connected to the central scattering volume $\Omega_{0}$ (red). Each terminal is associated, first, with a charge carrier reservoir $R_{s}$ defining the chemical potential $\mu_{s}$ (grey) of the carriers. Second, it is associated with a contact region $\Omega_{s}$ (blue) in which coherent scattering states are formed. In green we plot the interfaces $\Gamma_{s}$ between the $\Omega_{s}$ and $\Omega_{0}$ (solid) as well as the interfaces ${\bar{\Gamma }}_{s}$ between the $\Omega_{s}$ and $R_{s}$ (dashed). The coherence volume Ω of the scattering states comprises the set union of $\Omega_{0}$ and all $\Omega_{s}$. Here Γ is the surface of Ω excluding the ${\bar{\Gamma }}_{s}$ (magenta).

Our model for a multi-terminal system was described in Refs. [1,2]. It consists of a central quantum system located in the scattering volume $\Omega_{0}$ which is in contact with *N* terminals denoted with the index s=1…N (see Figure 1). In the scattering volume the potential acting on charge carriers can be arbitrary. For each terminal we assume the existence of, first, a reservoir $R_{s}$ for the charge carriers in which their chemical potential $\mu_{s}$ is defined and, second, a contact region $\Omega_{s}$ to the scattering volume in which coherent scattering states $\Psi^{s}$ are formed (see Equation (4)). The $\Psi^{s}$ are thus outgoing from this contact and they are coherent in the volume $\Omega =\Omega_{0}\cup \left(\cup_{s}\Omega_{s}\right)$. As illustrated in Figure 1 we define in each $\Omega_{s}$ a local coordinate system spanned by a triple or orthonormal basis vectors ${\vec{n}}_{s}$, ${\vec{t}}_{s}^{1}$, and ${\vec{t}}_{s}^{2}$ so that we can write

(1) $$\vec{r}={\vec{R}}_{s}+x_{s}{\vec{t}}_{s}^{1}+y_{s}{\vec{t}}_{s}^{2}+z_{s}{\vec{n}}_{s}\equiv {\vec{R}}_{s}+z_{s}{\vec{n}}_{s}+{\vec{r}}_{⊥;s},$$

where ${\vec{R}}_{s}$ points to the origin of the local coordinate system. The coordinate $z_{s}$ varies in the longitudinal direction and $x_{s}$ and $y_{s}$ in the two transverse directions. For the interface $\Gamma_{s}$ between $\Omega_{s}$ and $\Omega_{0}$ one has $z_{s}=0$ with $z_{s}$ growing towards the interior of the contact region. Furthermore, ${\vec{n}}_{s}$ is the surface normal vector to $\Gamma_{s}$. We require that the potential energy *V* of the charge carriers (electrons) in the contact regions takes the form

(2) $$V\left(\vec{r}\in \Omega_{s}\right)=V_{s}\left({\vec{r}}_{⊥;s}\right)-eU_{s}.$$

Here we assume that the reservoir $R_{1}$ is grounded with the chemical potential $\mu_{1}=\mu$. To each of the other reservoirs s≠1 a gate voltage $U_{s}$ is applied where we formally define $U_{1}=0$. Then one has $\mu_{s}=-eU_{s}+\mu$. As usual in the Landauer–Büttiker approach, the scattering states $\Psi^{s}$ which are formed in $\Omega_{s}$ are occupied according to the Fermi–Dirac distribution function with the chemical potential $\mu_{s}$. Furthermore, in $R_{s}$ the outgoing parts of the scattering states $\Psi^{s^{\prime }\neq s}$ arriving in *s* are absorbed completely, without any back-reflection.

Following further the theoretical framework of Landauer and Büttiker we start from the scattering solutions of the stationary Schrödinger equation

(3) $$\left[-\frac{\hbar^{2}}{2m^{*}}△+V\left(\vec{r}\right)-E\right]\Psi \left(\vec{r},E\right)=0$$

in the coherence region Ω. The relevant wave functions can be taken to vanish outside the coherence volume leading to the boundary condition $\Psi (\vec{r}\in \Gamma ,E)=0$ where Γ is the surface of Ω excluding the ${\bar{\Gamma }}_{s}$ (see Figure 1). The scattering solutions $\Psi^{sn}$ out-going from contact *s* can be written in each of the contacts $\Omega_{s^{\prime }}$ as

(4) $$\Psi^{sn}\left(\vec{r}\in \Omega_{s^{\prime }},E\right)=\exp\left(-ik_{sn}z_{s}\right)\Phi_{sn}\left({\vec{r}}_{⊥;s}\right)\delta_{s,s^{\prime }}+\sum_{n^{\prime }}S_{s^{\prime }n^{\prime },sn}\left(E\right)\exp\left(ik_{s^{\prime }n^{\prime }}z_{s^{\prime }}\right)\Phi_{s^{\prime }n^{\prime }}\left({\vec{r}}_{⊥;s^{\prime }}\right).$$

Here the transverse mode functions $\Phi_{sn}$ are the solutions of the eigenvalue problem

(5) $$\left[-\frac{\hbar^{2}}{2m^{*}}\Delta_{{\vec{r}}_{⊥;s}}+V_{s}\left({\vec{r}}_{⊥;s}\right)-E_{\nu }^{⊥}\right]\Phi_{\nu }\left({\vec{r}}_{⊥;s}\right)=0$$

defining the index of the transverse mode *n*, the composite mode index ν=(s,n), and $\Delta_{{\vec{r}}_{⊥;s}}=\partial^{2}/\partial x_{s}^{2}+\partial^{2}/\partial y_{s}^{2}$. The wave numbers of the harmonic waves in Equation (4) are given by

(6) $$k_{\nu }=\hbar^{-1}\sqrt{2m^{*}\left(E-E_{\nu }^{⊥}+eU_{s}\right)}.$$

The first factor on the right hand side of Equation (4) is the in-going part characterizing the scattering state. The second factor on the r.h.s. contains the out-going components which are determined by the S-matrix $S_{\nu^{\prime }\nu }$. In Section 3 we construct the S-matrix $S_{\nu^{\prime }\nu }$ in the R-matrix approach.

The total electric current $I_{s}$ in terminal *s* is calculated in Appendix A. We find

(7) $$I_{s}=\frac{2e}{h}\sum_{s^{\prime }}\int_{-\infty }^{\infty }dE\left[f\left(E-\mu_{s}\right)-f\left(E-\mu_{s^{\prime }}\right]\right)T_{s^{\prime }s}\left(E\right)$$

with the Fermi–Dirac distribution $f\left(x\right)={\left[e^{x/(k_{B}T)}+1\right]}^{-1}$, the elementary charge *e*, the current transmission sum

(8) $$T_{s^{\prime }s}\left(E\right)=\sum_{nn^{\prime }}\Theta \left(E-E_{sn}^{⊥}+eU_{s}\right)\Theta \left(E-E_{s^{\prime }n^{\prime }}^{⊥}+eU_{s^{\prime }}\right){\left|{\tilde{S}}_{s^{\prime }n^{\prime },sn}\left(E\right)\right|}^{2}=T_{ss^{\prime }}\left(E\right),$$

and the current S-matrix

(9) $${\tilde{S}}_{\nu^{\prime }\nu }=k_{\nu^{\prime }}^{1/2}S_{\nu^{\prime }\nu }k_{\nu }^{-1/2}.$$

## 3. Construction of the S-matrix with the R-matrix Method

We write the general solution of Equation (3) in each of the $\Omega_{s}$ in the form

(10) $$\Psi \left(\vec{r}\in \Omega_{s},E\right)=\sum_{n}\Psi_{sn}^{in}\exp\left(-ik_{sn}z_{s}\right)\Phi_{sn}\left({\vec{r}}_{⊥;s}\right)+\sum_{n}\Psi_{sn}^{out}\exp\left(ik_{sn}z_{s}\right)\Phi_{sn}\left({\vec{r}}_{⊥;s}\right).$$

Because of the linearity of the problem the S-matrix in Equation (4) can be defined as the linear mapping from the $\Psi^{in}$ onto the $\Psi^{out}$ of the form

(11) $$\Psi_{\nu }^{out}=\sum_{\nu^{\prime }}S_{\nu \nu^{\prime }}\Psi_{\nu^{\prime }}^{in}.$$

To construct $S_{\nu \nu^{\prime }}$ we expand the wave function in the scattering volume $\Omega_{0}$ in the orthonormal and complete set of Wigner–Eisenbud functions $\chi_{l}\left(\vec{r}\right)$,

(12) $$\Psi \left(\vec{r},E\right)=\sum_{l=1}^{\infty }a_{l}\left(E\right)\chi_{l}\left(\vec{r}\right)$$

with

(13) $$a_{l}\left(E\right)=\int_{\Omega_{0}}d\vec{r}\;\chi_{l}\left(\vec{r}\right)\Psi \left(\vec{r},E\right)$$

(see Appendix B). The Wigner–Eisenbud functions $\chi_{l}$ are the solutions of the Schrödinger equation

(14) $$\left[-\frac{\hbar^{2}}{2m^{*}}\Delta +V\left(\vec{r}\right)-E_{l}\right]\chi_{l}\left(\vec{r}\right)=0$$

in the domain $\Omega_{0}$. Here one imposes Wigner–Eisenbud boundary conditions, i.e., Neumann boundary conditions of vanishing normal derivative on the $\Gamma_{s}$,

(15) $$\frac{\partial \chi_{l}}{\partial {\vec{n}}_{s}}=0\;\;\;\;\mathrm{for}\;\;\;\;\;\vec{r}\in \Gamma_{s}$$

and Dirichlet boundary conditions on the remaining surface of $\Omega_{0}$ denoted with $\partial \Omega_{0}$ writing

(16) $$\chi_{l}=0\;\;\;\;\mathrm{for}\;\;\;\;\;\vec{r}\in \partial \Omega_{0}.$$

In Appendix B, we show that Wigner–Eisenbud energies $E_{l}$ are real and that the Wigner–Eisenbud functions $\chi_{l}\left(\vec{r}\right)$ can be chosen real. The normalization is taken as $\int_{\Omega_{0}}d\vec{r}{\left|\chi_{l}\left(\vec{r}\right)\right|}^{2}=1$. To calculate the expansion coefficients $a_{l}$ we multiply Equation (3) from the left with $\chi_{l}\left(\vec{r}\right)$ and Equation (14) from the left with $\Psi (\vec{r},E)$. Subtraction of the former equation from the latter and subsequent integration over the whole domain $\Omega_{0}$ yields with the second Green’s identity

(17) $$\begin{array}{ccc} \left(E-E_{l}\right)\int_{\Omega_{0}}d\vec{r}\;\chi_{l}\left(\vec{r}\right)\Psi \left(\vec{r},E\right) & = & -\frac{\hbar^{2}}{2m^{*}}\int_{\Omega_{0}}d\vec{r}\;\left[\chi_{l}\left(\vec{r}\right)△\Psi \left(\vec{r},E\right)-\Psi \left(\vec{r},E\right)△\chi_{l}\left(\vec{r}\right)\right]\\ & = & -\frac{\hbar^{2}}{2m^{*}}\sum_{s=1}^{N}\int_{\Gamma_{s}}d\Gamma_{s}{\vec{n}}_{s}\left[\chi_{l}\left(\vec{r}\right)\nabla \Psi \left(\vec{r},E\right)-\Psi \left(\vec{r},E\right)\nabla \chi_{l}\left(\vec{r}\right)\right]. \end{array}$$

In the area integration of Equation (17) as well as in the remaining area integrations over the $\Gamma_{s}$ we assume according to Equation (1) the parameterization $\vec{r}=\vec{r}\left(x_{s},y_{s}\right)={\vec{R}}_{s}+x_{s}{\vec{t}}_{s}^{1}+y_{s}{\vec{t}}_{s}^{2}$ of $\Gamma_{s}$ so that

(18) $$d\Gamma_{s}=dx_{s}dy_{s}\left|\frac{\partial \vec{r}}{\partial x_{s}}\times \frac{\partial \vec{r}}{\partial y_{s}}\right|=dx_{s}dy_{s}\left|{\vec{t}}_{s}^{1}\times {\vec{t}}_{s}^{2}\right|=dx_{s}dy_{s}.$$

Using in Equation (17) the notation

(19) $$\Psi^{S}\left(\vec{r}\in \Gamma_{s},E\right)=\frac{1}{m^{*}}{\vec{n}}_{s}\nabla \Psi \left(\vec{r}\in \Gamma_{s},E\right)$$

for the outward surface derivative, applying Equation (13) on the l. h. s., and inserting the boundary conditions for the Wigner–Eisenbud functions, one obtains

(20) $$a_{l}\left(E\right)=-\frac{\hbar^{2}}{2}\frac{1}{E-E_{l}}\sum_{s}\int_{\Gamma_{s}}d\Gamma_{s}\;\chi_{l}\left(\vec{r}\right)\;\Psi^{S}\left(\vec{r},E\right).$$

Returning to Equation (12) it follows that

(21) $$\Psi \left(\vec{r},E\right)=\sum_{s}\int_{\Gamma_{s}^{\prime }}d\Gamma_{s}^{\prime }\;R\left(\vec{r},{\vec{r}}^{\prime };E\right)\Psi^{S}\left({\vec{r}}^{\prime },E\right)$$

with

(22) $$R\left(\vec{r},{\vec{r}}^{\prime };E\right)=-\frac{\hbar^{2}}{2}\sum_{l=1}^{\infty }\frac{\chi_{l}\left(\vec{r}\right)\chi_{l}\left({\vec{r}}^{\prime }\right)}{E-E_{l}}.$$

For $\vec{r}\in \Gamma_{s}$ we write $\Psi \left(\vec{r}\left(x_{s},y_{s}\right),E\right)=\Psi \left({\vec{r}}_{⊥;s},E\right)$ and establish the expansion

(23) $$\Psi \left({\vec{r}}_{⊥;s},E\right)=\sum_{n}\Psi_{sn}\Phi_{sn}\left({\vec{r}}_{⊥;s}\right)$$

in the complete orthonormal and real function system of the $\Phi_{sn}$ with

(24) $$\Psi_{sn}=\int_{\Gamma_{s}}d\Gamma_{s}\;\Phi_{sn}\left({\vec{r}}_{⊥;s}\right)\Psi \left({\vec{r}}_{⊥;s},E\right).$$

An analogous expansion

(25) $$\Psi^{S}\left({\vec{r}}_{⊥;s},E\right)=\sum_{sn}\Psi_{sn}^{S}\Phi_{sn}\left({\vec{r}}_{⊥;s}\right)$$

holds for the surface derivative. Inserting the expansions Equations (23) and (25) in Equation (21) one obtains after a projection onto $\Phi_{\nu }$

(26) $$\Psi_{\nu }=\sum_{\nu^{\prime }}R_{\nu \nu^{\prime }}\Psi_{\nu^{\prime }}^{S}$$

with the R-matrix

(27) $$R_{\nu \nu^{\prime }}=\int_{\Gamma_{s}}d\Gamma_{s}\;\int_{\Gamma_{s^{\prime }}}d\Gamma_{s^{\prime }}^{\prime }\;\Phi_{\nu }\left({\vec{r}}_{⊥;s}\right)\Phi_{\nu^{\prime }}\left({\vec{r}}_{⊥;s^{\prime }}^{\prime }\right)R\left(\vec{r},{\vec{r}}^{\prime };E\right)=-\frac{\hbar^{2}}{2}\sum_{l=1}^{\infty }\frac{\chi_{l\nu }\chi_{l\nu^{\prime }}}{E-E_{l}},$$

where

(28) $$\chi_{l\nu }=\int_{\Gamma_{s}}d\Gamma_{s}\;\Phi_{\nu }\left({\vec{r}}_{⊥;s}\right)\chi_{l}\left(\vec{r}\right).$$

Inserting in Equation (26) $\Psi_{\nu }=\Psi_{\nu }^{in}+\Psi_{\nu }^{out}$ and ${\left(\Psi^{S}\right)}_{\nu }=-\left(ik_{\nu }/m^{*}\right)\left(\Psi_{\nu }^{in}-\Psi_{\nu }^{out}\right)$ one arrives at

$$\sum_{\nu^{\prime }}\left(\delta_{\nu \nu^{\prime }}-\frac{i}{m^{*}}R_{\nu \nu^{\prime }}k_{\nu^{\prime }}\right)\Psi_{\nu^{\prime }}^{out}=-\sum_{\nu^{\prime }}\left(\delta_{\nu \nu^{\prime }}+\frac{i}{m^{*}}R_{\nu \nu^{\prime }}k_{\nu^{\prime }}\right)\Psi_{\nu^{\prime }}^{in}.$$

Defining further a diagonal *k*-matrix $k_{\nu \nu^{\prime }}=\delta_{\nu \nu^{\prime }}k_{\nu^{\prime }}$ we formally write

(29) $$S=-\frac{1+\frac{i}{m^{*}}Rk}{1-\frac{i}{m^{*}}Rk}.$$

With the symmetrical current R-matrix

(30) $$\Omega_{\nu \nu^{\prime }}=k_{\nu }^{1/2}R_{\nu \nu^{\prime }}k_{\nu^{\prime }}^{1/2}$$

it follows for the current S-matrix in Equation (9) that

(31) $$\begin{array}{ccc} \tilde{S} & = & k^{1/2}Sk^{-1/2}=-k^{1/2}\left(1+iRk\right)k^{-1/2}k^{1/2}{\left(1-iRk\right)}^{-1}k^{-1/2}=-\left(1+i\Omega \right){\left(k^{-1/2}\right)}^{-1}{\left(1-iRk\right)}^{-1}{\left(k^{1/2}\right)}^{-1}\\ & = & -\left(1+i\Omega \right){\left[k^{1/2}\left(1-iRk\right)k^{-1/2}\right]}^{-1}=-\frac{1+i\Omega }{1-i\Omega }=1-\frac{2}{1-i\Omega }. \end{array}$$

Here we exploited that for three square matrices one has ${\left(ABC\right)}^{-1}=C^{-1}B^{-1}A^{-1}$. The current transmission matrix is thus seen to be symmetrical while the S-matrix is not symmetrical.

## 4. Transistor Model

**Figure 2:**
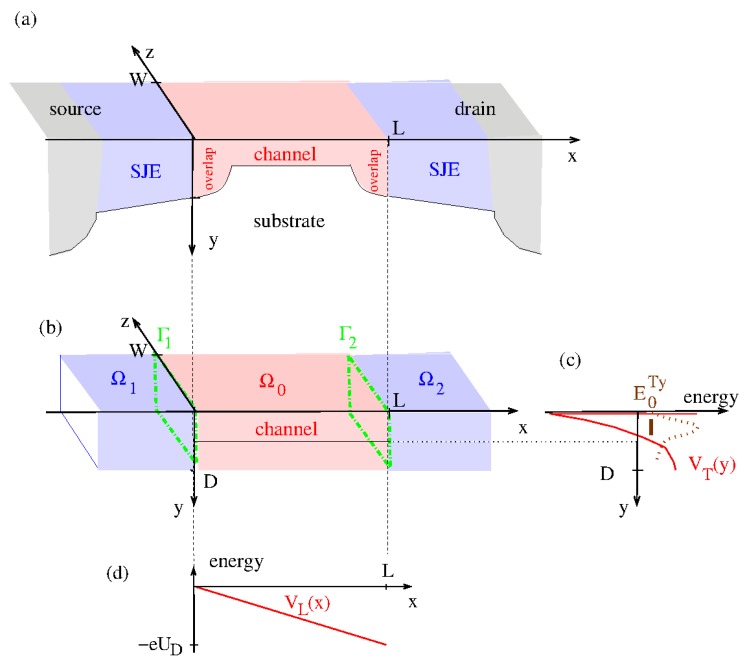
(**a**) Structure elements of a conventional nano-MOSFET: Source- and drain contact with shallow junction extensions SJEs, the latter in blue. In red the conduction channel and the overlap between conduction channel and SJE. The semiconductor-insulator interface is located at y=0. (**b**) Assignment of the above structure elements to the structure elements of the general multi-terminal system in Figure 1: The SJEs are associated with cubic contact regions $\Omega_{s}$. (**c**) In red: Transverse confinement potential $V_{T}\left(y\right)$ of the conduction channel in the separable ansatz for the potential in Equation (35). In brown the lowest subband energy $E_{0}^{Ty}$ in the channel confinement potential as defined in Equation (62) (solid) and the corresponding eigenfunction (dotted). (**d**) Linear drop of the applied drain voltage leading to a linear longitudinal potential $V_{L}\left(x\right)$ in Equation (35).

**Figure 3:**
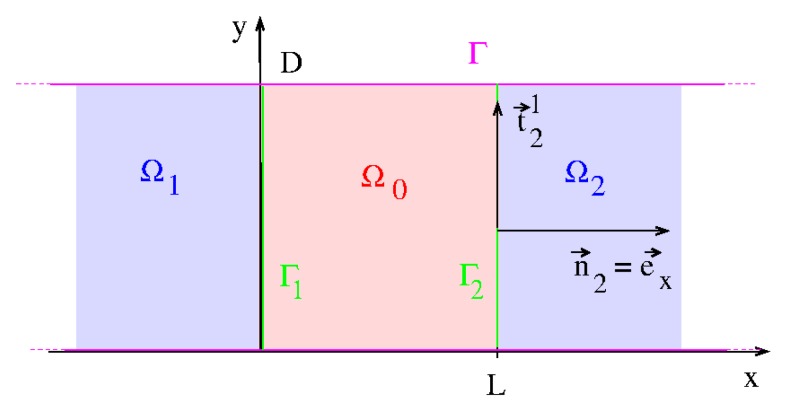
The two-terminal system in Figure 2b where the z-direction is omitted for simplicity. Axial contacts in x-direction: ${\vec{n}}_{2}$ points in *x*-direction, ${\vec{n}}_{1}$ in minus *x*-direction.

**Figure A1:**
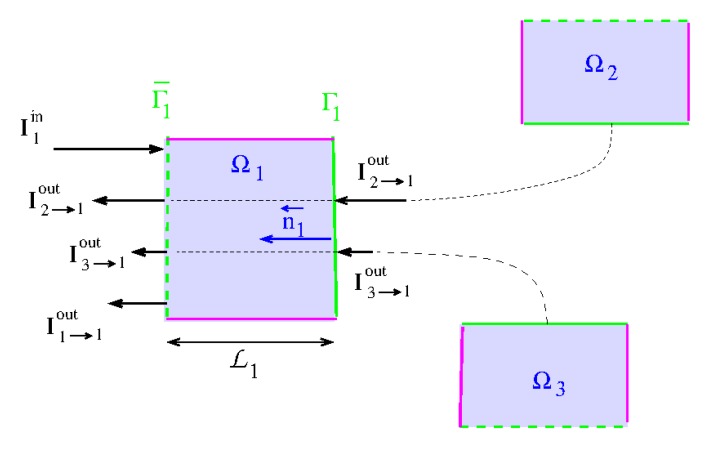
The formulation of Equation (A1) for contact s=1 in a N=3-terminal device at ${\bar{\Gamma }}_{1}$ (see also Figure 1). The current component directed in $-{\vec{n}}_{1}$-direction is $I_{1}^{in}$. The three current components in ${\vec{n}}_{1}$-direction are $I_{1\to 1}^{out}$, $I_{1\to 2}^{out}$, and $I_{1\to 3}^{out}$. Because there are no scattering processes in $\Omega_{1}$ it holds that $I_{1\to 2}^{out}$ and $I_{1\to 3}^{out}$ are the same in $\Gamma_{1}$ and ${\bar{\Gamma }}_{1}$ (see dashed horizontal lines).

The application of our model for a general multi-terminal system in Section 2 to a conventional n-channel nano-MOSFET is discussed in Ref. [1] (see in particular Figure 3 therein) and in Ref. [2]. Neglecting tunneling currents to the gate we here treat the transistor as a two-terminal device including only the source, s=1, and the drain, s=2. The relevant structure elements of a nano-MOSFET can be taken from Figure 2a depicting the heavily n-doped source- and drain contact, the shallow junction extensions (SJEs) of the contacts, the conduction channel in the p-substrate, and the overlap of the conduction channel with the SJE. The semiconductor-insulator interface is located at y=0. It is represented by a cut-off of the wave functions. The assignment of the structure elements of the nano-MOSFET to the structure elements of the general multi-terminal system in Figure 1 is shown in Figure 2b: The SJEs are assumed to be identical to having the depth *D*. The SJE of the source is then associated with the cubic contact region $\Omega_{1}$ with x≤0, 0≤y≤D, and 0≤z≤W. Here *W* is the width of the transistor. The SJE of the drain is associated with the cubic contact region $\Omega_{2}$ with x≥L, 0≤y≤D, and 0≤z≤W. Here $\Omega_{1}$ and $\Omega_{2}$ are semi-infinite corresponding to $L_{s}\to \infty$ (see Figure A1). The cubic scattering region $\Omega_{0}$ with 0≤x≤L, 0≤y≤D, and 0≤z≤W includes the conduction channel of length *L* and the overlap of the conduction channel with the SJEs. The interfaces $\Gamma_{s}$ are located at x=0 for s=1 and at x=L for s=2. The basis vectors of the local coordinate systems in Equation (1) are ${\vec{n}}_{1}=-{\vec{e}}_{x}$ and ${\vec{n}}_{2}={\vec{e}}_{x}$ for the outward normal vectors. Furthermore, we choose ${\vec{t}}_{1}^{1}={\vec{t}}_{2}^{1}={\vec{e}}_{y}$ and ${\vec{t}}_{1}^{2}={\vec{t}}_{1}^{2}={\vec{e}}_{z}$. The local coordinates are $z_{1}=-x$, $z_{2}=x-L$, $x_{1}=x_{2}=y$, and $y_{1}=y_{2}=z$. In Equation (2) we assume the simplest case $V_{s}=0$ renaming for $U_{2}=U_{D}$. We take the limit D→∞ as well as W→∞ so that electron gas in the heavily doped source and drain in $\Omega_{1}$ and $\Omega_{2}$ can be treated as a three dimensional free Fermi gas with the chemical potential

(32) $$\frac{\mu }{k_{B}T}=X_{1/2}\left[\frac{4}{3\sqrt{\pi }}{\left(\frac{E_{F}}{k_{B}T}\right)}^{3/2}\right]$$

where $X_{j}$ is the inverse function the Fermi–Dirac integral

(33) $$F_{1/2}\left(u\right)=\frac{1}{\Gamma (3/2)}\int_{0}^{\infty }dv\frac{v^{1/2}}{e^{v-u}+1}.$$

The Fermi energy above the bottom of the conduction band is given by

(34) $$E_{F}=\frac{\hbar^{2}}{2m^{e}}{\frac{3\pi^{2}N_{D}}{N_{V}}}^{2/3}$$

with the doping concentration $N_{D}$ in the contacts (full ionization of donors), the valley-degeneracy $N_{V}=6$ and the effective mass taken as $m^{e}={\left(m_{1}^{2}m_{2}\right)}^{1/3}=0.33m_{0}$. Here $m_{1}=0.19m_{0}$ and $m_{2}=0.98$ are the effective masses corresponding to the principle axes of the constant energy ellipsoids.

For the potential in the scattering area $\Omega_{0}$ we choose a separable form

(35) $$V\left(\vec{r}\in \Omega_{0}\right)=V_{T}\left(y\right)+V_{L}\left(x\right)$$

(see Figure 2c,d). Here the transverse potential $V_{T}$ is the confinement potential for the conduction channel of the transistor. A natural choice for $V_{T}$ is the confinement potential present in a simple MOS-structure without source- and drain contact as discussed in Refs. [50,51]. Then $V_{T}\left(y\right)$ corresponds to the potential determined in Equation (4) of [50]. As pointed out in Refs. [50,51] in the electron channel a strong lateral sub-band quantization exists so that only the lowest subband of the channel confinement potential with a bottom energy of $E_{0}^{Ty}$ corresponding to $E_{0}$ in Ref. [50] is occupied (see Figure 2c and Equation (62)). Here only the two constant energy ellipsoids with the heavy mass $m_{2}$ perpendicular to the (100)-interface are occupied. This leads to a valley degeneracy of $g_{v}=2$ in the channel and the effective mass entering (3) is the light mass $m^{*}=m_{1}$ [5]. The longitudinal potential $V_{L}$ arises from the applied drain voltage assumed to fall off linearly so that

(36) $$V_{L}\left(x\right)=-\frac{x}{L}eU_{G}.$$

The described transistor model has several special properties which can be used to simplify our general multi-terminal model described in Section 2:

P1 The transistor is treated as two-terminal system.
P2 *Axial contacts*: For all $\Gamma_{s}$ the surface normal vectors are aligned so that ${\vec{n}}_{s}=\pm \vec{n}$. For our transistor model ${\vec{n}}_{2}=-{\vec{n}}_{1}=\vec{n}={\vec{e}}_{x}$.
P3 *Global separability (see Figure 2b)*: In a system with axial contacts in $\vec{n}={\vec{e}}_{x}$-direction the potential in the scattering area $\Omega_{0}$ is the sum of a longitudinal potential $V_{L}\left(x\right)$ varying in $\vec{n}$-direction and transverse potential $V_{T}\left(y,z\right)$ varying in the two transverse directions. In the transistor model this separation is given in Equation (35).
P4 *Abrupt transition (see Figure 2c)*: An inspection of Equations (2) and (35) shows that in the general case the potentials in the contact regions and in the scattering volume come together to form an abrupt transition.
P5 *Planarity*: For a planar device one can define one or two global transverse coordinates valid in all $\Omega_{s}$ and in $\Omega_{0}$ on which the potential does not depend. In our transistor model one global transverse coordinate exists which is the width-coordinate *z*.
P7 *Single mode approximation:* One assumes strong transverse quantization in the scattering area. Then splitting of the transverse quantum levels induced by $V_{T}$ is so strong that only the lowest transverse level $E_{0}^{T}$ has to be taken into account.

As we will demonstrate in the next sections, on account of the listed special properties the R-matrix approach allows for a systematic reduction of the general theory for a multi-terminal device to a one-dimensional effective transistor model.

## 5. The R-matrix in a Separable Two-Terminal System

We consider a two-terminal system as in Figure 2b which fulfills the global separability condition P3 in Section 4 (see Figure 3). Inserting the separable potential Equation (35) in Equation (14) makes possible a product ansatz for the Wigner–Eisenbud functions

(37) $$\chi_{l}\left(\vec{r}\right)=\chi_{\lambda }\left(x\right)\phi_{k}\left(y,z\right)$$

with l=(k,λ). Here the transverse functions are defined by

(38) $$\left[-\frac{\hbar^{2}}{2m^{*}}\left(\frac{d^{2}}{dy^{2}}+\frac{d^{2}}{dz^{2}}\right)+V_{T}\left(y,z\right)-E_{k}^{T}\right]\phi_{k}\left(y,z\right)=0$$

with the boundary conditions

(39) $$\phi_{k}\left(0,z\right)=\phi_{k}\left(W,z\right)=\phi_{k}\left(y,0\right)=\phi_{k}\left(y,D\right)=0.$$

The longitudinal functions are the solutions of

(40) $$\left[-\frac{\hbar^{2}}{2m^{*}}\frac{d^{2}}{dx^{2}}+V_{L}\left(x\right)-E_{\lambda }^{L}\right]\chi_{\lambda }\left(x\right)=0$$

with the one-dimensional Wigner–Eisenbud boundary conditions

(41) $$\chi_{\lambda }^{\prime }\left(0\right)=\chi_{\lambda }^{\prime }\left(L\right)=0.$$

Upon insertion of Equation (37) in Equation (14) one obtains

(42) $$E_{l}=E_{\lambda }^{L}+E_{k}^{T}.$$

The product ansatz Equation (37) is permissible in the two-terminal system since the one-dimensional Wigner–Eisenbud boundary condition in Equation (41) is compatible with the general Wigner–Eisenbud boundary conditions in Equations (15) and (16). To construct the R-matrix with Equation (37) we write Equation (28) as

(43) $$\chi_{l\nu }=\int_{\Gamma_{s}}d\Gamma_{s}\;\Phi_{\nu }\left({\vec{r}}_{⊥;s}\right)\chi_{l}\left(\vec{r}\right)=\chi_{\lambda }\left(x_{s}\right)c_{ksn}$$

the overlap factor

(44) $$c_{ksn}=\int_{0}^{D}dy\int_{0}^{W}dz\phi_{k}\left(y,z\right)\Phi_{sn}\left(y,z\right).$$

The Equation (27) becomes

(45) $$R_{\nu \nu^{\prime }}\left(E\right)=-\frac{\hbar^{2}}{2}\sum_{\lambda k}\frac{c_{ksn}\chi_{\lambda }\left(x_{s}\right)c_{ks^{\prime }n^{\prime }}\chi_{\lambda }\left(x_{s^{\prime }}\right)}{E-E_{\lambda }^{L}-E_{k}^{T}}.$$

## 6. Effective Approximation and One-Dimensional Effective Scattering Problems

In effective approximation Equation (6) is simplified in the form

(46) $$k_{\nu }\left(E\right)∼\sqrt{\frac{2m^{*}}{\hbar^{2}}\left[E-E_{s}^{⊥0}+eU_{s}\right]}=k_{s}^{ef}\left(E\right),$$

where $E_{s}^{⊥0}$ is the smallest transverse mode energy, $E_{s}^{⊥0}={min}_{n}\left(E_{sn}^{⊥}\right)$. One then finds from Equations (30) and (45)

(47) $$\Omega_{sn,s^{\prime }n^{\prime }}=k_{sn}^{1/2}R_{sn,s^{\prime }n^{\prime }}k_{s^{\prime }n^{\prime }}^{1/2}=\sum_{k}c_{ksn}c_{ks^{\prime }n^{\prime }}\Omega_{ss^{\prime }}^{k}$$

with

(48) $$\Omega_{ss^{\prime }}^{k}=-\frac{\hbar^{2}}{2m^{*}}{\left(k_{s}^{ef}\right)}^{1/2}{\left(k_{s^{\prime }}^{ef}\right)}^{1/2}\sum_{\lambda }\frac{\chi_{\lambda }\left(x_{s}\right)\chi_{\lambda }\left(x_{s^{\prime }}\right)}{E-E_{\lambda }^{L}-E_{k}^{T}}.$$

The inversion of 1−iΩ in Equation (31) can now be carried out analytically with the result

(49) $${\left(\frac{1}{1-i\Omega }\right)}_{sns^{\prime }n^{\prime }}=\sum_{k}c_{ksn}c_{ks^{\prime }n^{\prime }}{\left(\frac{1}{1-i\Omega^{k}}\right)}_{ss^{\prime }}$$

(see Appendix C). Going back to Equation (31) one finds for $s\neq s^{\prime }$

(50) $$|{\tilde{S}}_{sn,s^{\prime }n^{\prime }}{|}^{2}={\left|{\left(\frac{2}{1-i\Omega }\right)}_{sn,s^{\prime }n^{\prime }}\right|}^{2}=\sum_{kk^{\prime }}c_{k,sn}c_{k,s^{\prime }n^{\prime }}c_{k^{\prime }sn}c_{k^{\prime }s^{\prime }n^{\prime }}{\left(\frac{2}{1-i\Omega^{k}}\right)}_{ss^{\prime }}{\left(\frac{2}{1-i\Omega^{k^{\prime }}}\right)}_{ss^{\prime }}^{*}.$$

With this relation Equation (A4) becomes with $I_{D}=I_{2}$

(51) $$I_{D}=\frac{2e}{h}\sum_{kk^{\prime }}\int_{-\infty }^{\infty }dEC_{kk^{\prime }}\left(E\right)\left[f\left(E-\mu \right)-f\left(E-\mu +eU_{D}\right)\right]{\left(\frac{2}{1-i\Omega^{k}}\right)}_{ss^{\prime }}{\left(\frac{2}{1-i\Omega^{k^{\prime }}}\right)}_{ss^{\prime }}^{*}$$

with the overlap matrix

(52) $$C_{kk^{\prime }}\left(E\right)=\sum_{n,n^{\prime }}c_{ksn}c_{ks^{\prime }n^{\prime }}c_{k^{\prime }sn}c_{k^{\prime }s^{\prime }n^{\prime }}\Theta \left[E-E_{sn}^{⊥}\right]\Theta \left[E-E_{s^{\prime }n^{\prime }}^{⊥}+eU_{D}\right].$$

In Appendix D, we demonstrate that instead of using Equation (48) to find $\left(1-i\Omega^{k}\right)$ with subsequent inversion one can calculate the matrices ${\left(1-i\Omega^{k}\right)}^{-1}$ occurring in Equation (51) according to

(53) $${\left(\frac{1}{1-i\Omega^{k}}\right)}_{21}=-\frac{\sqrt{k_{1}^{ef}k_{2}^{ef}}}{2}t^{ef}.$$

Here the $t^{ef}$ are the transmission coefficients resulting in an effective one-dimensional scattering problem associated with the 1d-Schrödinger equation

(54) $$\left[-\frac{\hbar^{2}}{2m^{*}}\frac{d^{2}}{dx^{2}}+V^{ef}\left(x\right)-E\right]\psi^{ef}\left(x\right)=0$$

with effective scattering potential

(55) $$V^{ef}\left(x\right)=\left\{\begin{array}{cc} E_{1}^{⊥0} & forx<0\\ E_{k}^{T}+V_{L}\left(x\right)\; & for0\leq x\leq L\\ E_{2}^{⊥0}-eU_{D} & forx>L. \end{array}\right.$$

Here the asymptotics of the source incident scattering states of the effective scattering problem associated with Equation (54) are given by

(56) $$\psi^{ef}\left(x<0\right)=e^{ik_{1}^{ef}x}e^{ik_{1}^{ef}x}+r^{ef}e^{-ik_{1}^{ef}x},$$

and

(57) $$\psi^{ef}\left(x\geq L\right)=t^{ef}e^{ik_{2}^{ef}\left(x-L\right)}.$$

Appendix E contains a simple, stable and fast recursive algorithm which we used to find the effective transmission coefficients $t^{ef}$. It is seen from Equation (55) that the quantum levels $E_{k}^{T}$ of the confinement potential in the conduction channel that arise in Equation (38) act as offsets in the effective potential.

## 7. Planar Systems and Supply Functions

In planar systems, the potential is taken as translationally invariant in the z-direction so that $V_{s}=V_{s}\left(y\right)$ and $V_{T}=V_{T}\left(y\right)$. For the interface regions $\Omega_{s}$ we insert in Equation (5)

(58) $$\Phi_{sn}\left(y,z\right)=\Phi_{sn_{y}n_{z}}\left(y,z\right)=\Phi_{sn_{y}}\left(y\right)\sqrt{\frac{2}{W}}\sin\left(\frac{n_{z}\pi }{W}z\right)$$

to find

(59) $$\left[-\frac{\hbar^{2}}{2m^{*}}\frac{d^{2}}{dy^{2}}+V_{s}\left(y\right)-E_{sn_{y}}^{⊥y}\right]\Phi_{sn_{y}}\left(y\right)=0$$

with $n=(n_{y},n_{z})$ and

(60) $$E_{sn}^{⊥}=E_{sn_{y}}^{⊥y}+\frac{\hbar^{2}}{2m^{*}}{\left(\frac{n_{z}\pi }{W}\right)}^{2}.$$

For the scattering region we insert in Equation (38)

(61) $$\phi_{k}\left(y,z\right)=\phi_{k_{y},k_{z}}\left(y,z\right)=\zeta_{k_{y}}\left(y\right)\sqrt{\frac{2}{W}}\sin\left(\frac{k_{z}\pi }{W}z\right)$$

to obtain

(62) $$\left[-\frac{\hbar^{2}}{2m^{*}}\frac{d^{2}}{dy^{2}}+V_{T}\left(y\right)-E_{k_{y}}^{Ty}\right]\zeta_{k_{y}}\left(y\right)=0,$$

with $k=(k_{y},k_{z})$ and

(63) $$E_{k}^{T}=E_{k_{y}}^{Ty}+\frac{\hbar^{2}}{2m^{*}}{\left(\frac{k_{z}\pi }{W}\right)}^{2}.$$

With Equations (58) and (62) the overlap factor in Equation (44) becomes

(64) $$c_{ksn}=\delta_{n_{z}k_{z}}\int_{0}^{W}dy\Phi_{sn_{y}}\left(y\right)\zeta_{k_{y}}\left(y\right)\equiv \delta_{n_{z}k_{z}}{\bar{c}}_{k_{y}sn_{y}}.$$

Furthermore from Equation (48) one has

(65) $$\Omega_{ss^{\prime }}^{k}=\Omega_{ss^{\prime }}^{k_{y},k_{z}}=-\frac{\hbar^{2}}{2m^{*}}{\left({\bar{k}}_{s^{\prime }}^{ef}\right)}^{1/2}{\left({\bar{k}}_{s}^{ef}\right)}^{1/2}\sum_{\lambda }\frac{\chi_{\lambda }\left(x_{s}\right)\chi_{\lambda }\left(x_{s^{\prime }}\right)}{E^{xy}-E_{\lambda }^{L}-E_{k_{y}}^{Ty}}\equiv {\bar{\Omega }}_{s^{\prime }s}^{k_{y}}\left(E^{xy}\right)$$

with the conserved energy in the xy-plane

(66) $$E^{xy}=E-\frac{\hbar^{2}}{2m^{*}}{\left(\frac{k_{z}\pi }{W}\right)}^{2},$$

and from Equation (46) $k_{s}^{ef}∼{\left[\left(2m^{*}/\hbar^{2}\right)\left(E^{xy}-E_{s}^{⊥y0}+eU_{s}\right)\right]}^{1/2}={\bar{k}}_{s}^{ef}\left(E^{xy}\right)$, where $E_{s}^{⊥y0}={min}_{n_{y}}\left(E_{sn_{y}}^{⊥y}\right)$. In Appendix F it is derived that

(67) $$I_{D}=\frac{2e}{h}\sum_{k_{y}k_{y}^{\prime }}\int_{-\infty }^{\infty }dE^{xy}C_{k_{y}k_{y}^{\prime }}\left(E^{xy}\right)\left[S\left(E^{xy}-\mu \right)-S\left(E^{xy}-\mu +eU_{D}\right)\right]{\left[\frac{2}{1-i{\bar{\Omega }}^{k_{y}}\left(E^{xy}\right)}\right]}_{ss^{\prime }}{\left[\frac{2}{1-i{\bar{\Omega }}^{k_{y}^{\prime }}\left(E^{xy}\right)}\right]}_{ss^{\prime }}^{*}$$

with wave function overlap

(68) $$C_{k_{y}k_{y}^{\prime }}\left(E^{xy}\right)=\sum_{n_{y}n_{y}^{\prime }}{\bar{c}}_{k_{y}sn_{y}}{\bar{c}}_{k_{y}s^{\prime }n_{y}^{\prime }}{\bar{c}}_{k_{y}^{\prime }sn_{y}}{\bar{c}}_{k_{y}^{\prime }s^{\prime }n_{y}^{\prime }}\Theta \left[E^{xy}-E_{sn_{y}}^{⊥y}\right]\Theta \left[E^{xy}-E_{s^{\prime }n_{y}^{\prime }}^{⊥y}+eU_{D}\right]$$

and the supply function

(69) $$S\left(\alpha \right)=\sum_{n_{z}}f\left[\alpha +\frac{\hbar^{2}}{2m^{*}}{\left(\frac{n_{z}\pi }{W}\right)}^{2}\right].$$

In the limit W→∞ we can write with $\Delta k_{z}=\pi /W$

(70) $$S\left(\alpha \right)=\frac{W}{\pi }\sum_{n_{z}}\Delta k_{z}f\left(\alpha +\frac{\hbar^{2}}{2m^{*}}k_{z}^{2}\right)\to \frac{W}{\pi }\int_{0}^{\infty }dk_{z}\frac{1}{e^{\frac{1}{k_{B}T}\left(\alpha +\frac{\hbar^{2}}{2m^{*}}{\left(n_{z}\Delta \right)}^{2}\right)}+1}.$$

Upon introducing

(71) $$y=\frac{1}{k_{B}T}\frac{\hbar^{2}}{2m^{*}}k_{z}^{2}\Rightarrow k_{z}=\sqrt{\frac{2m^{*}k_{B}T}{\hbar^{2}}}y^{1/2}\Rightarrow dk_{z}=\sqrt{\frac{2m^{*}k_{B}T}{\hbar^{2}}}\frac{1}{2}y^{-1/2}dy$$

it results that

(72) $$S\left(\alpha \right)=\frac{W}{\sqrt{\pi }}\sqrt{\frac{m^{*}k_{B}T}{2\hbar^{2}}}F_{-1/2}\left(-\frac{\alpha }{k_{B}T}\right).$$

Here the Fermi–Dirac-Integral is given by

(73) $$F_{j}\left(x\right)=\frac{1}{\Gamma (j+1)}\int_{0}^{\infty }dyy^{j}\frac{1}{1+e^{y-x}}$$

with $\Gamma \left(1/2\right)=\sqrt{\pi }$.

In Appendix D, we show that one can calculate the matrices ${\left(1-i{\bar{\Omega }}^{k_{y}}\right)}^{-1}$ in Equation (67) from the transmission coefficients resulting in a modified effective one-dimensional scattering problem. Here Equations (53)–(57) are substituted by

(74) $$\left[-\frac{\hbar^{2}}{2m^{*}}\frac{d^{2}}{dx^{2}}+{\bar{V}}^{ef}\left(x\right)-E^{xy}\right]{\bar{\psi }}^{ef}\left(x\right)=0,$$

for D→0

(75) $${\bar{V}}^{ef}\left(x\right)=\left\{\begin{array}{cc} 0 & forx<0\\ E_{k_{y}}^{Ty}+V_{L}\left(x\right)\; & for0\leq x\leq L\\ -eU_{D} & forx>L, \end{array}\right.$$

(76) $${\bar{\psi }}^{ef}\left(x<0\right)=e^{i{\bar{k}}_{1}^{ef}x}+r^{ef}e^{-i{\bar{k}}_{1}^{ef}x},$$

(77) $${\bar{\psi }}^{ef}\left(x\geq L\right)={\bar{t}}^{ef}e^{i{\bar{k}}_{2}^{ef}\left(x-L\right)},$$

and

(78) $$\sqrt{{\bar{k}}_{1}^{ef}{\bar{k}}_{2}^{ef}}{\bar{t}}_{1}^{k_{y}}=-{\left(\frac{2}{1-i{\bar{\Omega }}^{k_{y}}}\right)}_{21}.$$

## 8. Single-Mode Approximation and One-Dimensional Effective Model

As pointed out in Section 4, for a conventional nanotransistor only the lowest subband of the channel confinement potential with a bottom energy of $E_{0}^{Ty}$ resulting at $k_{y}=1$ is occupied (see Figure 2c and Equation (62)). Taking into account only $k_{y}=1$-terms Equation (67) becomes

(79) $$I_{D}=\frac{2N_{v}^{ch}e}{h}C\int_{0}^{\infty }dE^{xy}\left[S\left(E^{xy}-\mu \right)-S\left(E^{xy}-\mu +eU_{D}\right)\right]T^{ef}\left(E^{xy}\right)$$

with

(80) $$T^{ef}\left(E^{xy}\right)={\left[\frac{2}{1-i{\bar{\Omega }}^{1}\left(E^{xy}\right)}\right]}_{ss^{\prime }}{\left[\frac{2}{1-i{\bar{\Omega }}^{1}\left(E^{xy}\right)}\right]}_{ss^{\prime }}^{*}=k_{1}k_{2}{\left|t^{ef}\right|}^{2}$$

(compare with Equation (1) of Ref. [8]). Here we neglected in the wave function overlap the energy dependence, $C_{11}\left(E^{xy}\right)\to C\Theta \left(E^{xy}\right)$ and introduced the valley degeneracy of $N_{v}^{ch}=2$ in the n-type conduction channel.

As described in Section 7 the effective transmission coefficient ${\bar{t}}^{ef}$ is calculated from the source-incident scattering states of the 1d-Schrödinger Equation (74) with the effective scattering potential given by

(81) $${\bar{V}}^{ef}\left(x\right)=\left\{\begin{array}{cc} 0 & forx<0\\ V_{0}-eU_{D}\frac{x}{L}\; & for0\leq x\leq L\\ -eU_{D} & forx>L, \end{array}\right.$$

where set in Equation (75) $V_{L}\left(x\right)=-eU_{D}x/L$ (linear decrease of the drain voltage) and $E_{1}^{Ty}=V_{0}$. The parameter $V_{0}$ is interpretable as the effective height of the source-drain barrier. The parameters $V_{0}$ and *C* as well as *T* are adjusted to experiments in Refs. [6,7,8].

## 9. Summary

Starting from a basic description of quantum transport in a multi-terminal device in Landauer–Büttiker formalism in Refs. [1,2] we give a detailed derivation of all relevant formulas necessary to construct a one-dimensional effective model for a nanotransistor described in Refs. [6,7,8]. In this model, quantum transport in nano-FETs can be described quantitatively. Important device parameters can be extracted as the effective height of the source-drain barrier of the transistor, device heating, and the quality of the coupling between conduction channel and contacts.

## Appendix A. Derivation of the Formula for the Current

We calculate the total current $I_{s}$ in contact *s* starting from the decomposition

(A1) $$I_{s}=I_{s}^{in}-\sum_{s^{\prime }=1}^{N}I_{s^{\prime }\to s}^{out}$$

(see Figure A1). Here $I_{s}^{in}$ is the absolute value of the current in contact *s* created by the in-going parts of all scattering states

(A2) $$\Psi^{sn}\left(\vec{r},k\right)\equiv \Psi^{sn}\left(\vec{r},E=E_{sn}\left(k\right)\right)$$

where $E_{sn}\left(k\right)=\left(\hbar^{2}/2m^{*}\right)k^{2}+E_{sn}^{⊥}-eU_{s}$. Furthermore, $I_{s^{\prime }\to s}^{out}$ is the absolute value of the current in contact *s* created by the out-going parts of all scattering states $\Psi^{s^{\prime }n^{\prime }}\left(\vec{r},k^{\prime }\right)$ where $s^{\prime }$, $n^{\prime }$ and $k^{\prime }$ are arbitrary, thus including the case $s^{\prime }=s$ also. From current conservation one has

(A3) $$\sum_{s^{\prime }=1}^{N}I_{s\to s^{\prime }}^{out}=I_{s}^{in}.$$

From Equations (A1) and (A3) it results that

(A4) $$I_{s}=\sum_{s^{\prime }=1}^{N}\left(I_{s\to s^{\prime }}^{out}-I_{s^{\prime }\to s}^{out}\right)=\sum_{s^{\prime }}^{N,s^{\prime }\neq s}\left(I_{s^{\prime }\to s}^{out}-I_{s\to s^{\prime }}^{out}\right).$$

In Figure A1 the direction of the current contributions is given by the arrows for positive charge carriers. For n-type conduction the arrows have to be reversed.

### Appendix A.1. Current Contribution of a Single Scattering State

We decompose

(A5) $$I_{s\to s^{\prime }}^{out}=\sum_{nk}I_{s\to s^{\prime }}^{out,n}\left(k\right)$$

where $I_{s\to s^{\prime }}^{out,n}\left(k\right)$ is the absolute value of the current in contact $s^{\prime }$ created by the out-going part of the scattering state $\Psi^{sn}\left(\vec{r},k\right)$ given by

(A6) $$I_{s\to s^{\prime }}^{out,n}\left(k\right)=f_{FD}\left(E_{sn}\left(k\right)-\mu_{s}\right)\int_{\Gamma_{s^{\prime }}}d\Gamma_{s^{\prime }}j^{out}\left(\vec{r}\right).$$

Here for $\vec{r}\in \Omega_{s^{\prime }}$

(A7) $$j^{out}\left(\vec{r}\right)={\vec{n}}_{s^{\prime }}\vec{j}\left(\vec{r}\right)$$

with

(A8) $$\vec{j}\left(\vec{r}\right)=\frac{e\hbar }{2m^{*}i}{\left|N\right|}^{2}\left[\Psi^{sn}{\left(\vec{r},k\right)}^{*}\nabla \Psi^{sn}\left(\vec{r},k\right)-\Psi^{sn}\left(\vec{r},k\right)\nabla \Psi^{sn}{\left(\vec{r},k\right)}^{*}\right]$$

since from Equation (A4) the case $s=s^{\prime }$ can be excluded. In Equation (A8) N is the continuum normalization constant to be constructed in Equation (A17). The issue of the k-summation in Equation (A5) is addressed in Appendix A.2. From Equation (4) we have for $\vec{r}\in \Omega_{s^{\prime }}$

(A9) $$\Psi^{sn}\left(\vec{r},k\right)=\sum_{n^{\prime }}S_{s^{\prime }n^{\prime },sn}\exp\left(ik_{s^{\prime }n^{\prime }}z_{s^{\prime }}\right)\Phi_{s^{\prime }n^{\prime }}\left(x_{s^{\prime }},y_{s^{\prime }}\right)$$

and

(A10) $${\vec{n}}_{s^{\prime }}\nabla \Psi^{sn}\left(\vec{r},k\right)=\frac{\partial }{\partial z_{s^{\prime }}}\Psi^{sn}\left(\vec{r},k\right)=\sum_{n^{\prime }}S_{s^{\prime }n^{\prime },sn}ik_{s^{\prime }n^{\prime }}\exp\left(ik_{s^{\prime }n^{\prime }}z_{s^{\prime }}\right)\Phi_{s^{\prime }n^{\prime }}\left(x_{s^{\prime }},y_{s^{\prime }}\right)$$

where

(A11) $$k_{s^{\prime }n^{\prime }}=\hbar^{-1}\sqrt{2m^{*}\left(E_{sn}\left(k\right)-E_{s^{\prime }n^{\prime }}^{⊥}+eU_{s^{\prime }}\right)}.$$

The area integration in Equation (A6) leads to

(A12) $$I_{s\to s^{\prime }}^{out,n}\left(k\right)=\frac{e\hbar }{m^{*}}{\left|N\right|}^{2}f_{FD}\left(E_{sn}\left(k\right)-\mu_{s}\right)\sum_{n^{\prime }}^{\mathrm{prop}}k_{s^{\prime }n^{\prime }}{\left|S_{s^{\prime }n^{\prime },sn}\right|}^{2}$$

where the index prop restricts the summation to propagating waves with real, positive $k_{s^{\prime }n^{\prime }}$.

### Appendix A.2. Summation Over Scattering States

To calculate $I_{s\to s^{\prime }}^{out}$ according to Equation (A5) one sums Equation (A12) over all scattering states, i. e. over all *n* and *k*, according to their occupation in the form

(A13) $$I_{s\to s^{\prime }}^{out}=\sum_{n}\sum_{j}D_{k}\Delta kI_{s\to s^{\prime }}^{out,n}\left(k\right)\to D_{k}\sum_{n}\int_{0}^{\infty }dkI_{s\to s^{\prime }}^{out,n}\left(k\right),$$

with the discretization

(A14) $$k\to k_{j}=j\Delta k>0\;for\;j\in N^{0}.$$

Here the constant $D_{k}$ is the density of scattering states in k-space which we will address in Equation (A16). The width of the k-intervals Δk is assumed to be small so that the k-integration can be replaced by a Riemann sum. As usual, one determines $D_{k}$ and N by assigning to each normalized propagating solution of the Schrödinger equation in $\Omega_{s}$ of the form

(A15) $$Ne^{ikz}\Phi_{sn}\left(y,z\right)$$

the normalized scattering state $N\Psi^{sn}\left(\vec{r},k\right)$ which has the in-going part in Equation (A15). As is well-known, this corresponds to the expectation that each in-coming particle is represented by a wave-package. When the particle is located deeply in the interior of $\Omega_{s}$ it does not ’feel’ the quantum system and it can be equivalently represented by a superposition of the plane waves in Equation (A15) or the in-going part of the normalized scattering states $N\Psi^{sn}\left(\vec{r},k\right)$. The plane waves solutions Equation (A15) can now be counted and normalized introducing artificial boundary conditions in $\Omega_{s}$ in the interval $0\leq z_{s}<L_{s}$ (see Figure A1) so that

(A16) $$k_{j}=j\frac{2\pi }{L_{s}}\Rightarrow D_{k}=\frac{2}{2\pi /L_{s}}$$

where in the last step we included a simple spin-degeneracy factor of two. The normalization then follows from

(A17) $$1={\left|N\right|}^{2}\int_{0}^{L_{s}}dz{\left|e^{ikz}\right|}^{2}\Rightarrow N=\frac{1}{\sqrt{L_{s}}}$$

Upon insertion in Equation (A13) one finds with ${\left|N\right|}^{2}D_{k}=1/\pi$

(A18) $$I_{s\to s^{\prime }}^{out}=\frac{e\hbar }{m^{*}}\frac{1}{\pi }\sum_{nn^{\prime }}^{\mathrm{prop}}\int_{0}^{\infty }dkf\left[E_{sn}\left(k\right)-\mu_{s}\right]k_{s^{\prime }n^{\prime }}\left(k\right){\left|S_{s^{\prime }n^{\prime },sn}\left(E_{sn}\left(k\right)\right)\right|}^{2}.$$

Substituting further

(A19) $$E=E_{sn}\left(k\right)=\left(\hbar^{2}/2m^{*}\right)k^{2}+E_{sn}^{⊥}-eU_{s}$$

so that

(A20) $$k=\sqrt{\frac{2m^{*}}{\hbar^{2}}}{\left(E_{sn}\left(k\right)-E_{sn}^{⊥}+eU_{s}\right)}^{1/2}\Rightarrow \frac{dk}{dE}=\sqrt{\frac{m^{*}}{2\hbar^{2}}}{\left(E_{sn}\left(k\right)-E_{sn}^{⊥}+eU_{s}\right)}^{-1/2}=\frac{m^{*}}{\hbar^{2}}k{\left(E\right)}^{-1}.$$

With dk→(dk/dE)dE one obtains from Equation (A18)

(A21) $$\begin{array}{ccc} I_{s\to s^{\prime }}^{out} & = & \frac{1}{\pi }\frac{e\hbar }{m^{*}}\frac{m^{*}}{\hbar^{2}}\sum_{n}^{prop}\int_{E_{⊥}^{n}-eU_{s}}^{\infty }dE\sum_{n^{\prime }}^{\mathrm{prop}}f\left(E-\mu_{s}\right)k_{s^{\prime }n^{\prime }}\left(E\right){\left|S_{s^{\prime }n^{\prime },sn}\left(E\right)\right|}^{2}k_{sn}{\left(E\right)}^{-1}\\ & = & \frac{2e}{h}\sum_{n}\int_{-\infty }^{\infty }dE\Theta \left(E-E_{sn}^{⊥}+eU_{s}\right)\sum_{n^{\prime }}f\left(E-\mu_{s}\right)k_{s^{\prime }n^{\prime }}\left(E\right){\left|S_{s^{\prime }n^{\prime },sn}\left(E\right)\right|}^{2}k_{sn}{\left(E\right)}^{-1}\Theta \left(E-E_{s^{\prime }n^{\prime }}^{⊥}+eU_{s^{\prime }}\right)\\ & = & \frac{2e}{h}\int_{-\infty }^{\infty }dEf\left(E-\mu_{s}\right)\sum_{nn^{\prime }}\Theta \left(E-E_{sn}^{⊥}+eU_{s}\right)\Theta \left(E-E_{s^{\prime }n^{\prime }}^{⊥}+eU_{s^{\prime }}\right)k_{s^{\prime }n^{\prime }}\left(E\right){\left|S_{s^{\prime }n^{\prime },sn}\left(E\right)\right|}^{2}k_{sn}{\left(E\right)}^{-1}\\ & = & \frac{2e}{h}\int_{-\infty }^{\infty }dEf\left(E-\mu_{s}\right)T_{s^{\prime }s}\left(E\right) \end{array}$$

with the current transmission sum

(A22) $$\begin{array}{ccc} T_{s^{\prime }s}\left(E\right) & = & \sum_{nn^{\prime }}\Theta \left(E-E_{sn}^{⊥}+eU_{s}\right)\Theta \left(E-E_{s^{\prime }n^{\prime }}^{⊥}+eU_{s^{\prime }}\right)k_{s^{\prime }n^{\prime }}\left(E\right){\left|S_{s^{\prime }n^{\prime },sn}\left(E\right)\right|}^{2}k_{sn}{\left(E\right)}^{-1}\\ & = & \sum_{nn^{\prime }}\Theta \left(E-E_{sn}^{⊥}+eU_{s}\right)\Theta \left(E-E_{s^{\prime }n^{\prime }}^{⊥}+eU_{s^{\prime }}\right){\left|{\tilde{S}}_{s^{\prime }n^{\prime },sn}\left(E\right)\right|}^{2}=T_{ss^{\prime }}\left(E\right). \end{array}$$

The symmetry relation ${\tilde{S}}_{s^{\prime }n^{\prime },sn}={\tilde{S}}_{sn,s^{\prime }n^{\prime }}$ is shown in Equation (31). Because $T_{s^{\prime }s}=T_{ss^{\prime }}$ one finds from Equation (A21) from

(A23) $$I_{s}=\left(I_{s\to s^{\prime }}^{out}-I_{s^{\prime }\to s}^{out}\right)=\frac{2e}{h}\int_{-\infty }^{\infty }dE\left[f\left(E-\mu_{s}\right)-f\left(E-\mu_{s}^{\prime }\right]\right)T_{s^{\prime }s}\left(E\right).$$

## Appendix B. Properties of the Wigner–Eisenbud Problem

(1) *Hermiticity:*
  We take two functions $\psi_{1}\left(\vec{r}\right)$ and $\psi_{2}\left(\vec{r}\right)$ obeying the Wigner–Eisenbud boundary conditions Equations (15) and (16), i. e., with the Neumann boundary conditions $\left[\partial \psi /\partial {\vec{n}}_{s}\right]\left(\vec{r}\in \Gamma_{s}\right)=0$ and Dirichlet boundary condition $\psi (\vec{r}\in \partial \Omega_{0})=0$. From second Green’s theorem it follows directly that
  (A24) $$\int_{\Omega_{0}}dv\left(\psi_{1}^{*}\Delta \psi_{2}-\psi_{2}\Delta \psi_{1}^{*}\right)=\sum_{s}\int_{\Gamma_{s}}d\Gamma_{s}{\vec{n}}_{s}\left(\psi_{1}^{*}\nabla \psi_{2}-\psi_{2}\nabla \psi_{1}^{*}\right)=0.$$
  As desired, one immediately obtains the hermicity condition
  (A25) $$\int_{\Omega_{0}}dv\left[\psi_{1}^{*}\left(H\psi_{2}\right)-\psi_{2}{\left(H\psi_{1}\right)}^{*}\right]=-\frac{\hbar^{2}}{2m}\int_{\Omega_{0}}dv\left(\psi_{1}^{*}\Delta \psi_{2}-\psi_{2}\Delta \psi_{1}^{*}\right)=0.$$
(2) *The Wigner–Eisenbud energies are real:*
  The Wigner–Eisenbud functions are the eigenfunctions of *H*,
  (A26) $$\left[H-E_{l}\right]\chi_{l}=0$$
  obeying Wigner–Eisenbud conditions. Setting in Equation (A25) $\psi_{1}=\psi_{2}=\chi_{l}$ it follows that
  (A27) $$0=\int_{\Omega_{0}}dv\chi_{l}^{*}H\chi_{l}-\int_{\Omega_{0}}dv\chi_{l}{\left[H\chi_{l}\right]}^{*}=\left[E_{l}-E_{l}^{*}\right]\underset{\in R^{+}}{\underset{︸}{\int_{\Omega_{0}}dv\chi_{l}^{*}\chi_{l}}}.$$
(3) *The Wigner–Eisenbud functions can be chosen real:*
  Since the $E_{l}$ are real the complex conjugate of Equation (A26) is given by
  (A28) $$\left[H-E_{l}\right]\chi_{l}^{*}=0.$$
  From the sum of Equations (A26) and (A28) one obtains
  (A29) $$\left[H-E_{l}\right]\left(\chi_{l}+\chi_{l}^{*}\right)=0\Rightarrow \left[H-E_{l}\right]Re\left(\chi_{l}\right)=0$$
  and from the difference
  (A30) $$\left[H-E_{l}\right]\left(\chi_{l}-\chi_{l}^{*}\right)=0\Rightarrow \left[H-E_{l}\right]Im\left(\chi_{l}\right)=0.$$
  Therefore, if a complex function $\chi_{l}$ is a solution of Equation (A26) then $\chi_{l}^{*}$ is a solution too and one can choose instead of $\chi_{l}$ two real solutions $Re(\chi_{l})$ and $Im(\chi_{l})$.
(4) *The Wigner–Eisenbud functions are orthogonal:*
  For two Wigner–Eisenbud functions with different energies $E_{l}\neq E_{l^{\prime }}$ we write
  (A31) $$\left[H-E_{l^{\prime }}\right]\chi_{l^{\prime }}=0.$$
  Setting in Equation (A25) $\psi_{1}^{*}=\psi_{1}=\chi_{l^{\prime }}$ and $\psi_{2}^{*}=\psi_{2}=\chi_{l}$
  (A32) $$0=\int_{\Omega_{0}}dv\left[\chi_{l^{\prime }}\left(H\chi_{l}\right)-\chi_{l}{\left(H\chi_{l^{\prime }}\right)}^{*}\right]=\underset{\neq 0}{\underset{︸}{\left(E_{l}-E_{l^{\prime }}\right)}}\int_{\Omega_{0}}dv\chi_{l^{\prime }}\chi_{l}.$$
  For degenerate Wigner–Eisenbud functions $E_{l}=E_{l^{\prime }}$ two orthogonal linear combinations can be constructed with standard methods.
(5) *Completeness:*
  As described in (1) the operator *H* is hermitic, it is second order in the derivatives and linear. Then the set of its eigenfunctions $\chi_{l}$, the Wigner–Eisenbud functions, is complete. Thus, with (3) and (4) the $\chi_{l}$ can be chosen as a complete, real, orthonormal function system.

## Appendix C. Verification of Equation (49)

We verify this equation explicitly:

(A33) $$\begin{array}{cc} & {\left[\left(1-i\Omega \right)\left(\frac{1}{1-i\Omega }\right)\right]}_{sns^{\prime }n^{\prime }}=\sum_{s^{\prime \prime }n^{\prime \prime }}{\left(1-i\Omega \right)}_{sn,s^{\prime \prime }n^{\prime \prime }}{\left(\frac{1}{1-i\Omega }\right)}_{s^{\prime \prime }n^{\prime \prime },s^{\prime }n^{\prime }}\\ = & \sum_{s^{\prime \prime }n^{\prime \prime }k^{\prime }}\left(\delta_{sn,s^{\prime \prime }n^{\prime \prime }}-i\Omega_{sn,s^{\prime \prime }n^{\prime \prime }}\right)c_{k^{\prime }s^{\prime \prime }n^{\prime \prime }}c_{k^{\prime }s^{\prime }n^{\prime }}{\left(\frac{1}{1-i\Omega^{k^{\prime }}}\right)}_{s^{\prime \prime }s^{\prime }}\\ = & \sum_{s^{\prime \prime }n^{\prime \prime }kk^{\prime }}\left(c_{ksn}c_{ksn^{\prime \prime }}\delta_{s,s^{\prime \prime }}-ic_{ksn}c_{ks^{\prime \prime }n^{\prime \prime }}\Omega_{ss^{\prime \prime }}^{k}\right)c_{k^{\prime },s^{\prime \prime }n^{\prime \prime }}c_{k^{\prime },s^{\prime }n^{\prime }}{\left(\frac{1}{1-i\Omega^{k^{\prime }}}\right)}_{s^{\prime \prime }s^{\prime }}\\ = & \sum_{s^{\prime \prime }n^{\prime \prime }kk^{\prime }}c_{ksn}\underset{\delta_{kk^{\prime }}}{\underset{︸}{c_{ks^{\prime \prime }n^{\prime \prime }}c_{k^{\prime }s^{\prime \prime }n^{\prime \prime }}}}c_{k^{\prime },s^{\prime }n^{\prime }}\left(\delta_{s,s^{\prime \prime }}-i\Omega_{ss^{\prime \prime }}^{k}\right){\left(\frac{1}{1-i\Omega^{k^{\prime }}}\right)}_{s^{\prime \prime }s^{\prime }}\\ = & \sum_{s^{\prime \prime }kk^{\prime }}c_{ksn}\delta_{kk^{\prime }}c_{k^{\prime }s^{\prime }n^{\prime }}{\left(1-i\Omega^{k}\right)}_{ss^{\prime \prime }}{\left(\frac{1}{1-i\Omega^{k^{\prime }}}\right)}_{s^{\prime \prime }s^{\prime }}\\ = & \sum_{s^{\prime \prime }k}c_{ksn}c_{ks^{\prime }n^{\prime }}{\left(1-i\Omega^{k}\right)}_{ss^{\prime \prime }}{\left(\frac{1}{1-i\Omega^{k}}\right)}_{s^{\prime \prime }s^{\prime }}=\delta_{ss^{\prime }}\sum_{k}\underset{\delta_{nn^{\prime }}}{\underset{︸}{c_{ksn}c_{ksn^{\prime }}}}=\delta_{ss^{\prime }}\delta_{nn^{\prime }}. \end{array}$$

Here we applied the relations in under-braces

(A34) $$\sum_{n}c_{ksn}c_{k^{\prime }sn}=\delta_{kk^{\prime }}\;\mathrm{and}\;\sum_{k}c_{ksn}c_{ksn^{\prime }}=\delta_{nn^{\prime }}.$$

To derive the first relation we formulate the completeness of the $\Phi_{sn}$ and the $\phi_{k}$ writing

(A35) $$\sum_{n}\Phi_{sn}\left(y,z\right)\Phi_{sn}\left(y^{\prime },z^{\prime }\right)=\delta \left(x-x^{\prime }\right)\delta \left(y-y^{\prime }\right)=\sum_{k}\phi_{k}\left(y,z\right)\phi_{k}\left(y^{\prime },z^{\prime }\right).$$

Projection onto $\phi_{k^{\prime \prime }}\left(y,z\right)$ and $\phi_{k^{\prime }}\left(y^{\prime },z^{\prime }\right)$ yields immediately

(A36) $$\sum_{n}c_{k^{\prime \prime }sn}c_{k^{\prime }sn}=\delta_{k^{\prime \prime },k^{\prime }}.$$

The second relation in Equation (A34) is derived by inserting in the orthogonality relation

(A37) $$\int_{0}^{D}dy\int_{0}^{W}dz\Phi_{sn}\left(y,z\right)\Phi_{sn^{\prime }}\left(y,z\right)=\delta_{nn^{\prime }}$$

the expansion $\Phi_{sn}\left(y,z\right)=\sum_{k}c_{ksn}\phi_{k}\left(y,z\right)$. It is seen that

(A38) $$\delta_{nn^{\prime }}=\sum_{kk^{\prime }}c_{ksn}c_{k^{\prime }sn^{\prime }}\int_{0}^{D}dy\int_{0}^{W}dz\phi_{k}\left(y,z\right)\phi_{k^{\prime }}\left(y,z\right)=\sum_{kk^{\prime }}c_{ksn}c_{k^{\prime }sn^{\prime }}\delta_{kk^{\prime }}=\sum_{k}c_{ksn}c_{ksn^{\prime }}.$$

## Appendix D. R-matrix Theory in One Dimension

We define the Wigner–Eisenbud functions in one dimension as the solutions of the hermitic eigenvalue problem

(A39) $$\left[-\frac{\hbar^{2}}{2m^{*}}\frac{d^{2}}{dx^{2}}+V^{ef}\left(x\right)-E_{\lambda }^{ef}\right]\chi_{\lambda }\left(x\right)=0$$

with the effective 1d-scattering potential given in Equation (55) and von-Neumann boundary conditions

(A40) $$\chi_{\lambda }^{\prime }\left(0\right)=\chi_{\lambda }^{\prime }\left(L\right)=0.$$

A comparison with Equation (40) yields identical eigenfunctions $\chi_{\lambda }$ and eigenenergies shifted by $E_{k}^{T}$,

(A41) $$E_{\lambda }^{ef}=E_{\lambda }^{L}+E_{k}^{T}.$$

The $\chi_{\lambda }$ constitute a complete orthonormal system in which the scattering states $\psi^{ef}$ in Equation (54) can be expanded in the domain x∈[0,L]. One has

(A42) $$\psi^{ef}\left(x\right)\equiv \psi \left(x\right)=\sum_{\lambda =1}^{\infty }a_{\lambda }\chi_{\lambda }\left(x\right),$$

where

(A43) $$a_{\lambda }=\int_{0}^{L}\psi \left(x\right)\chi_{\lambda }\left(x\right)dx.$$

The left-multiplication of Equation (54) with $\chi_{\lambda }\left(x\right)$ and left-multiplication of Equation (A39) with ψ(x) leads after integration to

$$-\frac{\hbar^{2}}{2m}\int_{0}^{L}dx\;\left[\chi_{\lambda }\left(x\right)\frac{d^{2}}{dx^{2}}\psi \left(x\right)-\psi \left(x\right)\frac{d^{2}}{dx^{2}}\chi_{\lambda }\left(x\right)\right]=\left(E-E_{\lambda }^{L}-E_{k}^{T}\right)\underset{a_{\lambda }}{\underset{︸}{\int_{0}^{L}dx\;\psi \left(x\right)\chi_{l}\left(x\right)}}.$$

Partial integration on the left side and application of the von-Neumann boundary conditions Equation (A40) leads to

(A44) $$-\frac{\hbar^{2}}{2m}\left[\chi_{\lambda }\left(L\right)\frac{d\psi }{dx}\left(L\right)-\chi_{\lambda }\left(0\right)\frac{d\psi }{dx}\left(0\right)\right]=\left(E-E_{\lambda }^{L}-E_{k}^{T}\right)a_{\lambda }.$$

Following Equation (19) we introduce the outward directed normal derivatives

(A45) $$\psi^{S}\left(0\right)=-\frac{1}{m^{*}}\frac{d\psi }{dx}{|}_{x=0}\;\mathrm{as}\;\mathrm{well}\;\mathrm{as}\;\psi^{S}\left(L\right)=\frac{1}{m^{*}}\frac{d\psi }{dx}{|}_{L}$$

and obtain

(A46) $$-\frac{\hbar^{2}}{2}\frac{\chi_{\lambda }\left(0\right)\psi^{S}\left(0\right)+\chi_{\lambda }\left(L\right)\psi^{S}\left(L\right)}{E-E_{\lambda }^{L}-E_{k}^{T}}=a_{\lambda }.$$

Upon multiplication with $\chi_{\lambda }\left(x\right)$ and summation λ one finds

(A47) $$\psi \left(x\right)=\sum_{\lambda =1}^{\infty }a_{\lambda }\left(E\right)\chi_{\lambda }\left(x\right)=R^{k}\left(x,0\right)\psi_{S}\left(0\right)+R^{k}\left(x,L\right)\psi_{S}\left(L\right),$$

with

(A48) $$R^{k}\left(x,x^{\prime }\right)=-\frac{\hbar^{2}}{2}\sum_{\lambda =1}^{\infty }\frac{\chi_{\lambda }\left(x\right)\chi_{\lambda }\left(x^{\prime }\right)}{E-E_{\lambda }^{L}-E_{k}^{T}}.$$

From evaluation of this equation for $x_{1}=0$ and $x_{2}=L$ one finds in correspondence to Equation (26)

(A49) $$\psi \left(x_{s}\right)=\sum_{s^{\prime }}R_{ss^{\prime }}^{k}\psi^{S}\left(x_{s}\right)\Rightarrow \vec{\psi }=R^{k}{\vec{\psi }}^{S},$$

where we define the 2×2 R-matrix

(A50) $$R_{ss^{\prime }}^{k}=-\frac{\hbar^{2}}{2m^{*}}\sum_{\lambda =1}^{\infty }\frac{\chi_{\lambda }\left(x_{s}\right)\chi_{\lambda }\left(x_{s^{\prime }}\right)}{E-E_{\lambda }^{L}-E_{k}^{T}}$$

and the two-component vectors

(A51) $${\left(\vec{\psi }\right)}_{s}=\psi \left(x_{s}\right)\;\mathrm{and}\;{\left({\vec{\psi }}^{S}\right)}_{s}=\psi^{S}\left(x_{s}\right).$$

A comparison of Equation (A50) with Equation (48) yields

(A52) $$\Omega_{ss^{\prime }}^{k}={\left(k_{s}^{ef}\right)}^{1/2}{\left(k_{s^{\prime }}^{ef}\right)}^{1/2}R_{ss^{\prime }}^{k}.$$

We now proceed as in Equation (10) and decompose the general solution of the wave function in the contacts in an in-going part and an out-going part, $\psi \left(x\right)=\psi^{in}\left(x\right)+\psi^{out}\left(x\right)$, where

(A53) $$\psi^{in}\left(x\right)=\left\{\begin{array}{cc} \psi_{1}^{in}e^{ik_{1}^{ef}x} & \mathrm{for}\;x<0\\ \; & \;\\ \psi_{2}^{in}e^{-ik_{2}^{ef}\left(x-L\right)} & \mathrm{for}\;x>L \end{array}\right.$$

and

(A54) $$\psi^{out}\left(x\right)=\left\{\begin{array}{cc} \psi_{1}^{out}e^{-ik_{1}^{ef}x} & \mathrm{for}\;x<0\\ \; & \;\\ \psi_{2}^{out}e^{ik_{2}^{ef}\left(x-L\right)} & \mathrm{for}\;x>L. \end{array}\right.$$

As in Equation (11), the S-matrix is the linear mapping of the in-going part onto the out-going part

(A55) $${\vec{\psi }}^{out}=S^{k}{\vec{\psi }}^{in}$$

with the two-component vector

(A56) $${\left({\vec{\psi }}^{in}\right)}_{s}=\psi_{s}^{in}\;\mathrm{and}\;{\left({\vec{\psi }}^{out}\right)}_{s}=\psi_{s}^{out}.$$

The source-incident scattering states are associated with $\psi_{1}^{in}=1$ and $\psi_{2}^{in}=0$, $\psi_{1}^{out}=S_{11}^{k}=r_{1}^{k}$ and $\psi_{2}^{out}=S_{21}^{k}=t_{1}^{k}$. The drain-incident scattering states are associated with $\psi_{1}^{in}=0$ and $\psi_{2}^{in}=1$, $\psi_{1}^{out}=S_{12}^{k}=t_{2}^{k}$ and $\psi_{2}^{out}=S_{22}^{k}=r_{2}^{k}$. One finds the relation between S-matrix and the transmission- and reflection coefficients

(A57) $$\left(\begin{array}{cc} S_{11}^{k} & S_{12}^{k}\\ S_{21}^{k} & S_{22}^{k} \end{array}\right)=\left(\begin{array}{cc} r_{1}^{k} & t_{2}^{k}\\ t_{1}^{k} & r_{2}^{k} \end{array}\right).$$

Using Equations (A53) and (A54) it results for x≤0

(A58) $$\frac{d\psi^{in}\left(x\right)}{dx}=ik_{1}^{ef}\psi^{in}\left(x\right)\;\mathrm{and}\;\frac{d\psi^{out}\left(x\right)}{dx}=-ik_{1}^{ef}\psi^{out}\left(x\right),$$

and for x≥L

(A59) $$\begin{array}{c} \frac{d\psi^{in}\left(x\right)}{dx}=-ik_{2}^{ef}\psi^{ein}\left(x\right)\;\mathrm{and}\;\frac{d\psi^{out}\left(x\right)}{dx}=ik_{2}^{ef}\psi^{out}\left(x\right). \end{array}$$

It follows that

(A60) $${\vec{\psi }}_{S}=ik^{ef}{\vec{\psi }}^{out}-ik^{ef}{\vec{\psi }}^{in},$$

with the diagonal wave number matrix ${\left(k^{ef}\right)}_{ss^{\prime }}=\delta_{ss^{\prime }}k_{s}^{ef}$. From Equation (A49), $\vec{\psi }={\vec{\psi }}^{in}+{\vec{\psi }}^{out}$, and Equation (A60) it follows that

(A61) $$\left(iR^{k}k^{ef}-1\right){\vec{\psi }}^{out}=\left(iR^{k}k^{ef}+1\right){\vec{\psi }}^{in}.$$

A comparison with Equation (A55) leads to

(A62) $$S^{k}=-\frac{1+iR^{k}k^{ef}}{1-iR^{k}k^{ef}}.$$

For the current matrix we find with Equation (A52)

(A63) $${\tilde{S}}^{k}={\left(k^{ef}\right)}^{1/2}S^{k}{\left(k^{ef}\right)}^{-1/2}=\frac{1+i{\left(k^{ef}\right)}^{1/2}R^{k}{\left(k^{ef}\right)}^{1/2}}{1-i{\left(k^{ef}\right)}^{1/2}R^{k}{\left(k^{ef}\right)}^{1/2}}-\frac{1+i\Omega^{k}}{1-i\Omega^{k}}=\frac{-2+1-i\Omega^{k}}{1-i\Omega^{k}}=1-\frac{2}{1-i\Omega^{k}}.$$

It is now decisive that with the definition of the $R^{k}$ in Equation (A50) it results that

(A64) $${\left(\Omega^{k}\right)}_{ss^{\prime }}={\left(k_{s}^{ef}\right)}^{1/2}R_{ss^{\prime }}^{k}{\left(k_{s^{\prime }}^{ef}\right)}^{1/2}=-\frac{\hbar^{2}}{2m^{*}}{\left(k_{s}^{ef}\right)}^{1/2}{\left(k_{s^{\prime }}^{ef}\right)}^{1/2}\sum_{\lambda =1}^{\infty }\frac{\chi_{\lambda }\left(x_{s}\right)\chi_{\lambda }\left(x_{s^{\prime }}\right)}{E-E_{\lambda }^{L}-E_{k}^{T}}$$

identical with Equation (48). From Equation (A57) one has

(A65) $${\tilde{S}}_{21}^{k}=\sqrt{k_{1}^{ef}k_{2}^{ef}}t_{1}^{k}=-{\left(\frac{2}{1-i\Omega^{k}}\right)}_{21}.$$

In Section 6, we identified $\psi^{ef}$ with the source-incident scattering state characterized through the asymptotic in Equations (76) and (77). Therefore we identify $t_{1}^{k}=t^{ef}$ and Equation (A70) becomes Equation (53).

In Equation (65) we define for the planar system in Section 7

(A66) $${\bar{\Omega }}_{s^{\prime }s}^{k_{y}}\left(E^{xy}\right)=-\frac{\hbar^{2}}{2m^{*}}{\left({\bar{k}}_{s^{\prime }}^{ef}\right)}^{1/2}{\left({\bar{k}}_{s}^{ef}\right)}^{1/2}\sum_{\lambda }\frac{\chi_{\lambda }\left(x_{s}\right)\chi_{\lambda }\left(x_{s^{\prime }}\right)}{E^{xy}-E_{\lambda }^{L}-E_{k_{y}}^{Ty}}\equiv {\bar{\Omega }}_{s^{\prime }s}^{k_{y}}\left(E^{xy}\right)$$

with the conserved energy in the xy-plane

(A67) $$E^{xy}=E-\frac{\hbar^{2}}{2m^{*}}{\left(\frac{k_{z}\pi }{W}\right)}^{2}$$

and $k_{sn}\left(E\right)={\bar{k}}_{s}^{ef}\left(E^{xy}\right)$. Comparing Equation (A66) with Equation (A64) one can adopt the result Equation (A70) for the planar system if one identifies $k↔k_{y}$ $E↔E^{xy}$, $k_{s}^{ef}\left(E\right)↔{\bar{k}}_{s}^{ef}\left(E^{xy}\right)$, and $E_{k}^{T}↔E_{k_{y}}^{Ty}$. This way an effective one-dimensional scattering problem associated with the 1d-Schrödinger equation

(A68) $$\left[-\frac{\hbar^{2}}{2m^{*}}\frac{d^{2}}{dx^{2}}+V^{ef}\left(x\right)-E^{xy}\right]\psi^{ef}\left(x\right)=0$$

results with the effective scattering potential in the limit W→0 given by

(A69) $$V^{ef}\left(x\right)=\left\{\begin{array}{cc} 0 & \mathrm{for}\;x<0\\ E_{k_{y}}^{Ty}+V_{L}\left(x\right)\; & \mathrm{for}\;0\leq x\leq L\\ -eU_{D} & \mathrm{for}\;x>L. \end{array}\right.$$

The transmission coefficients ${\bar{t}}_{1}^{k_{y}}$ of the source-incident scattering functions of Equation (A68) yield

(A70) $${\bar{t}}_{1}^{k_{y}}={\tilde{S}}_{21}^{k_{y}}=\sqrt{k_{1}^{ef}k_{2}^{ef}}{\bar{t}}_{1}^{k_{y}}=-{\left(\frac{2}{1-i{\bar{\Omega }}^{k_{y}}}\right)}_{21}.$$

## Appendix E. Numerical Evaluation of the Transmission Coefficients in One Dimension

In the finite difference method, the one-dimensional Schrödinger equation

(A71) $$\left[-\frac{\hbar^{2}}{2m}\frac{d^{2}}{dx^{2}}+V\left(x\right)-E\right]\psi \left(x,E\right)=0$$

becomes

(A72) $$\psi_{n+1}+\psi_{n-1}-2\psi_{n}+\frac{2m\Delta^{2}}{\hbar^{2}}\left(E-V_{n}\right)\psi_{n}=0.$$

Here we discretize the real axis in the form $x\to x_{n}=n\Delta$ with Δ→0. Requiring L=NΔ one has N+1 grid points in the scattering area 0≤x≤L. Furthermore, we introduce $V\left(x\right)\to V\left(x_{n}\right)\equiv V_{n}$, $\psi \left(x,E\right)\to \psi \left(x_{n},E\right)\equiv \psi_{n}$, and $d^{2}/\left(dx^{2}\right)\to \left(\psi_{n+1}+\psi_{n-1}-2\psi_{n}\right)/\Delta^{2}$. In view of Equation (81) we assume the asymptotics $V_{n<0}=0$ (source) and $V_{n>N}=-eU_{D}$ (drain). The source-incident scattering states then follow the asymptotic

(A73) $$\psi_{n}=\left\{\begin{array}{cc} r\exp\left(-ik_{1}n\Delta \right)+\exp\left(ik_{1}n\Delta \right)\; & \mathrm{for}\;n<0\\ \; & \;\\ t\exp\left(ik_{2}\left(n-N\right)\Delta \right)\; & \mathrm{for}\;n>N \end{array}\right.$$

with $k_{s}=\sqrt{2m(E-V_{s})}/\hbar^{2}$ with $V_{1}=0$ and $V_{2}=-eU_{D}$. To construct the source-incident scattering states we transform Equation (A72) for $\phi_{n}=\psi_{n}/t$ into a downward recursion

(A74) $$\phi_{n-1}=-\phi_{n+1}+\left[2+\frac{2m\Delta^{2}}{\hbar^{2}}\left(V_{n}-E\right)\right]\phi_{n}.$$

For $\phi_{n}$ one has

(A75) $$\phi_{n}=\left\{\begin{array}{cc} \frac{r}{t}\exp\left(-ik_{1}Ln/N\right)+\frac{1}{t}\exp\left(ik_{1}Ln/N\right)\; & \mathrm{for}\;n<0\\ \; & \;\\ \exp\left(ik_{2}L\left(n-N\right)/N\right)\; & \mathrm{for}\;n>N. \end{array}\right.$$

with the known asymptotic on the drain side

(A76) $$\phi_{n>N}=\exp\left(ik_{2}L\left(n-N\right)/N\right).$$

The the downward recursion Equation (A74) is started with, for example,

(A77) $$\phi_{N+2}=\exp\left(2ik_{2}L/N\right)\;\;\;\;\mathrm{and}\;\;\;\;\;\phi_{N+3}=\exp\left(3ik_{2}L/N\right)$$

to construct $\phi_{n}$ in the entire range. Especially one obtains

(A78) $$\phi_{-2}=\frac{1}{t}\exp\left(-2ik_{1}L/N\right)+\frac{r}{t}\exp\left(2ik_{1}L/N\right)$$

and

(A79) $$\phi_{-3}=\frac{1}{t}\exp\left(-3ik_{1}L/N\right)+\frac{r}{t}\exp\left(3ik_{1}L/N\right).$$

The Equations (A78) and (A79) represent two linear equations for the two unknown *t* and *r*. One finds

(A80) $$t=\frac{\exp\left(-3ik_{1}L/N\right)-\exp\left(-ik_{1}L/N\right)}{\phi_{-3}-\exp\left(ik_{1}L/N\right)\phi_{-2}}.$$

## Appendix F. Derivation of the Supply Function

Starting from Equation (51) it follows that

(A81) $$\begin{array}{ccc} I_{D} & = & \frac{2e}{h}\sum_{nn^{\prime }kk^{\prime }}\int_{-\infty }^{\infty }dE\left[f\left(E-\mu \right)-f\left(E-\mu +eU_{D}\right)\right]c_{ksn}c_{ks^{\prime }n^{\prime }}c_{k^{\prime }sn}c_{k^{\prime }s^{\prime }n^{\prime }}\\ & & \;\;\times {\left(\frac{2}{1-i\Omega^{k}}\right)}_{ss^{\prime }}{\left(\frac{2}{1-i\Omega^{k^{\prime }}}\right)}_{ss^{\prime }}^{*}\Theta \left[E-E_{sn}^{⊥}\right]\Theta \left[E-E_{s^{\prime }n^{\prime }}^{⊥}+eU_{D}\right]\\ & = & \frac{2e}{h}\sum_{n_{y}n_{z}n_{y}^{\prime }n_{z}^{\prime }k_{y}k_{z}k_{y}^{\prime }k_{z}^{\prime }}\int_{-\infty }^{\infty }dE\left[f\left(E-\mu \right)-f\left(E-\mu +eU_{D}\right)\right]{\bar{c}}_{k_{y}sn_{y}}\delta_{n_{z}k_{z}}{\bar{c}}_{k_{y}s^{\prime }n_{y}^{\prime }}\delta_{n_{z}k_{z}^{\prime }}{\bar{c}}_{k_{y}^{\prime }sn_{y}}\delta_{n_{z}^{\prime }k_{z}}{\bar{c}}_{k_{y}^{\prime }s^{\prime }n_{y}^{\prime }}\delta_{n_{z}^{\prime }k_{z}^{\prime }}\\ & & \;\;\times {\left(\frac{2}{1-i\Omega^{k_{y}k_{z}}}\right)}_{ss^{\prime }}{\left(\frac{2}{1-i\Omega^{k_{y}^{\prime }k_{z}^{\prime }}}\right)}_{ss^{\prime }}^{*}\Theta \left[E-E_{sn_{y}n_{z}}^{⊥}\right]\Theta \left[E-E_{s^{\prime }n_{y}^{\prime }n_{z}^{\prime }}^{⊥}+eU_{D}\right]\\ & = & \frac{2e}{h}\sum_{n_{y}n_{z}n_{y}^{\prime }k_{y}k_{y}^{\prime }}\int_{-\infty }^{\infty }dE\left[f\left(E-\mu \right)-f\left(E-\mu +eU_{D}\right)\right]{\bar{c}}_{k_{y}sn_{y}}{\bar{c}}_{k_{y}s^{\prime }n_{y}^{\prime }}{\bar{c}}_{k_{y}^{\prime }sn_{y}}{\bar{c}}_{k_{y}^{\prime }s^{\prime }n_{y}^{\prime }} \end{array}$$

(A82) $$\begin{array}{ccc} & & \;\;\times {\left(\frac{2}{1-i\Omega^{k_{y}n_{z}}}\right)}_{ss^{\prime }}{\left(\frac{2}{1-i\Omega^{k_{y}^{\prime }n_{z}}}\right)}_{ss^{\prime }}^{*}\Theta \left[E-E_{sn_{y}n_{z}}^{⊥}\right]\Theta \left[E-E_{s^{\prime }n_{y}^{\prime }n_{z}}^{⊥}+eU_{D}\right]\\ & = & \frac{2e}{h}\sum_{n_{z}n_{y}n_{y}^{\prime }k_{y}k_{y}^{\prime }}\int_{-\infty }^{\infty }dE^{xy}\left[f\left(E^{xy}+\frac{\hbar^{2}}{2m^{*}}{\left(\frac{n_{z}\pi }{W}\right)}^{2}-\mu \right)-f\left(E^{xy}+\frac{\hbar^{2}}{2m^{*}}{\left(\frac{n_{z}\pi }{W}\right)}^{2}-\mu +eU_{D}\right)\right]{\bar{c}}_{k_{y}sn_{y}}{\bar{c}}_{k_{y}s^{\prime }n_{y}^{\prime }}{\bar{c}}_{k_{y}^{\prime }sn_{y}}{\bar{c}}_{k_{y}^{\prime }s^{\prime }n_{y}^{\prime }} \end{array}$$

(A83) $$\begin{array}{ccc} & & \;\;\times {\left[\frac{2}{1-i{\bar{\Omega }}^{k_{y}}\left(E^{xy}\right)}\right]}_{ss^{\prime }}{\left[\frac{2}{1-i{\bar{\Omega }}^{k_{y}^{\prime }}\left(E^{xy}\right)}\right]}_{ss^{\prime }}^{*}\Theta \left[E^{xy}-E_{sn_{y}}^{⊥y}\right]\Theta \left[E^{xy}-E_{s^{\prime }n_{y}^{\prime }}^{⊥y}+eU_{D}\right]\\ & = & \frac{2e}{h}\sum_{k_{y}k_{y}^{\prime }}\int_{-\infty }^{\infty }dE^{xy}{\bar{C}}_{k_{y}k_{y}^{\prime }}\left(E^{xy}\right)\left[S\left(E^{xy}-\mu \right)-S\left(E^{xy}-\mu +eU_{D}\right)\right]{\left[\frac{2}{1-i{\bar{\Omega }}^{k_{y}}\left(E^{xy}\right)}\right]}_{ss^{\prime }}{\left[\frac{2}{1-i{\bar{\Omega }}^{k_{y}^{\prime }}\left(E^{xy}\right)}\right]}_{ss^{\prime }}^{*} \end{array}$$

with

(A84) $$C_{k_{y}k_{y}^{\prime }}\left(E^{xy}\right)=\sum_{n_{y}n_{y}^{\prime }}{\bar{c}}_{k_{y}sn_{y}}{\bar{c}}_{k_{y}s^{\prime }n_{y}^{\prime }}{\bar{c}}_{k_{y}^{\prime }sn_{y}}{\bar{c}}_{k_{y}^{\prime }s^{\prime }n_{y}^{\prime }}\Theta \left[E^{xy}-E_{sn_{y}}^{⊥y}\right]\Theta \left[E^{xy}-E_{s^{\prime }n_{y}^{\prime }}^{⊥y}+eU_{D}\right]$$

and

(A85) $$S\left(\alpha \right)=\sum_{n_{z}}f\left[\alpha +\frac{\hbar^{2}}{2m^{*}}{\left(\frac{n_{z}\pi }{W}\right)}^{2}\right].$$

Going over from Equation (A81) to Equation (A82) we made use of Equation (65) with $k_{z}=n_{z}$.
